# Lipids Modulate the Increase of BK Channel Calcium Sensitivity by the β1 Subunit

**DOI:** 10.1371/journal.pone.0107917

**Published:** 2014-09-25

**Authors:** Chunbo Yuan, Cristina Velázquez-Marrero, Alexandra Bernardo, Steven N. Treistman

**Affiliations:** Institute of Neurobiology, University of Puerto Rico, San Juan, Puerto Rico; Indiana University School of Medicine, United States of America

## Abstract

Co-expression of the auxiliary β1 subunit with the pore forming α subunit of BK dramatically alters apparent calcium sensitivity. Investigation of the mechanism underlying the increase in calcium sensitivity of BK in smooth muscle has concentrated on the energetic effect of β1′s interaction with α. We take a novel approach, exploring whether β1 modification of calcium sensitivity reflects altered interaction between the channel protein and surrounding lipids. We reconstituted hSlo BK α and BK α+β1 channels into two sets of bilayers. One set contained POPE with POPS, POPG, POPA and POPC, where the length of acyl chains is constant, but surface charge differs. The second set is a series of neutral bilayers formed from DOPE with phosphatidylcholines (PCs) of varying acyl chain lengths: C (14∶1), C (18∶1), C (22∶1) and C (24∶1), and with brain sphingomyelin (SPM), in which surface charge is constant, but bilayer thickness varies. The increase in calcium sensitivity caused by the β1 subunit was preserved in negatively charged lipid bilayers but not in neutral bilayers, indicating that modification of apparent Ca^2+^ sensitivity by β1 is modulated by membrane lipids, requiring negatively charged lipids in the membrane. Moreover, the presence of β1 reduces BK activity in thin bilayers of PC 14∶1 and thick bilayers containing SPM, but has no significant effect on activity of BK in PC 18∶1, PC 22∶1 and PC 24∶1 bilayers. These data suggest that auxiliary β1 subunits fine-tune channel gating not only through direct subunit-subunit interactions but also by modulating lipid-protein interactions.

## Introduction

Large conductance, Ca^2+^-activated K^+^ channels (BK) play a crucial role in the regulation of arterial tone, where they facilitate a negative feedback mechanism that opposes vasoconstriction [1], [2]. Intravascular pressure increases arterial tone by a complex process that includes membrane depolarization and the subsequent elevation of cytoplasmic Ca^2+^ via voltage-dependent Ca^2+^ channels. This global increase in Ca^2+^ leads to vasoconstriction, but also triggers localized Ca^2+^ release events (termed Ca^2+^ sparks) from ryanodine receptors on the smooth muscle sacroplasmic reticulum. The Ca^2+^ release events activate nearby BKs, creating a hyperpolarizing K+ current, known as spontaneous transient outward currents (STOCs), which opposes further constriction. The greater Ca^2+^ sensitivity of smooth muscle BK than the BK channels in many other tissues is attributable to the co-expression of β1 subunit with the pore-forming α subunit [3], [4], [5], [6]. The importance of the β1 has been demonstrated in β1 knockout mice, which display reduced BK channel opening, increased vascular tone, and hypertension [1].

The mechanisms by which β1 enhances the apparent Ca^2+^ sensitivity of the BK channel have been the subject of a number of studies [3], [4], [5], [6], [7], [8], [9], [10], [11], [12], [13]. Since β1 causes a negative voltage shift of the conductance-voltage (G–V) relationship, the effect of β1 was initially described as an “increase in apparent Ca^2+^ sensitivity” [14]. β1 was shown to facilitate the gating of BK channels by acting through the Ca^2+^ but not the Mg^2+^ activating mechanism [4], and the Slo1 tail domains, but not the Ca^2+^ bowl were required for β1 to increase the apparent Ca^2+^ sensitivity [4]. Later, it was found that β1 effects on BK may not be due exclusively to changes in Ca^2+^ binding equilibrium [5], [8], [15]. Further investigation demonstrated that β1 has two major effects on channel gating energetics: 1) β1 reduces the intrinsic closed-open equilibrium and 2) shifts the open channel voltage sensor activation to negative membrane potentials [12].

Studies to date have focused largely on interactions between the α subunit and the β1 subunit. Interactions between membrane lipids and the channel protein, and the influence of these lipids on β1 modulation of BK function have not been adequately addressed. Both α and β1 subunits of BK channels are transmembrane proteins, and their function relies on the lipid bilayer in which they are imbedded. The pore-forming α subunit has seven transmembrane segments (S0–S6) and a large intracellular C-terminal containing the Ca^2+^ bowl. S1–S4 forms a voltage sensor domain, and S5–S6 forms the ion conduction pore. The auxiliary β subunit has two transmembrane segments (TM1 and TM2) and a large extracellular loop [16]. We have shown that membrane lipids modulate the basal function, such as channel conductance and channel gating of BK, [17], [18] and alter the ethanol sensitivity of the channel [19], [20]. We hypothesize that membrane lipids could also play a critical role in shaping β1 modulation of BK function. In this study, we tested our hypothesis by reconstituting BK channels composed of hSlo α and α+β into two series of lipid bilayers that independently vary the surface charge and the thickness of the bilayers. We found that the increase of apparent Ca^2+^ sensitivity by the β1 subunit is more dramatic in bilayers composed of negatively charged lipids than in bilayers made of neutral lipids, suggesting lipid modulation of the increase of apparent Ca^2+^ sensitivity in the presence of β1. In bilayers made of neutral lipids of increasing acyl chain length (from PC 14∶1 to PC 24∶1), β1 has little effect on the single channel activities in bilayers of PC 18∶1, PC 22∶1 and PC 24∶1, but reduces channel activity in the thin bilayer of PC 14∶1 and the thick bilayer of SPM. Once again, this suggests an important role for the lipid environment in the effects of β1 on BK Ca^2+^ sensitivity.

## Materials and Methods

### Materials

**Figure 1:**
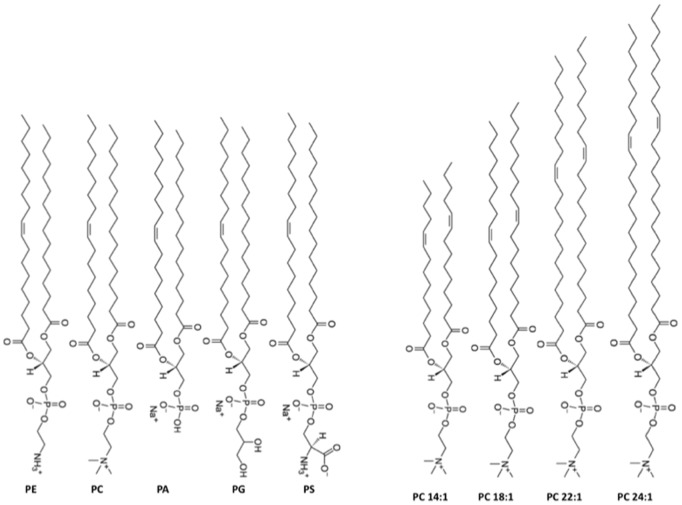
Molecular structure of the lipids used in this study. The left panel shows lipids having identical acyl chains but with different polar headgroups. The right panel shows lipids having identical polar headgroups but different acyl chain length.

POPE, POPS, POPG, POPA, POPC, DOPE, brain SPM and PCs (from C14∶1, to C24∶1) were obtained from Avanti Polar Lipids (Alabaster, AL). The molecular structure of these lipids is shown in Fig. 1. They were used without further purification. Decane and salts were from Aldrich Chem. Co. Inc. (St. Louis, MO). All aqueous solutions were prepared with 18.3 MΩ.cm Milli-Q water.

### Membrane preparation

The hSlo α and hSlo α+β1 channels for all experiments were derived from 2 stable cell lines. The HEK/***α***-1.2 cell line stably expresses the human BK channel *α*-subunit splice variant, hbr1 derived from brain [21]. The hSlo α+β1 channels were derived from the HEK/*αβ*-B7 cell line stably expressing hbr 1, and the h*β*_1_-subunit. Both cell lines were a gift from Peter Ahring. Stably transfected HEK-293 cells were grown in artificial medium and membrane fragments were prepared using a protocol developed for COS cells with some slight modifications as described elsewhere [22].

### Electrophysiology

HEK 293 cells stably expressing hSlo α and hSlo α+*β1* channels were cultured in 35-mm culture dishes until 45–60% confluence was reached. Cells were washed for 20 min in high calcium (2.2 mM) Locke’s solution followed by 10-min wash in intracellular recording solution (1, 5 or 10 µM free calcium) prior to recording. Single-channel recordings were performed in excised inside-out membrane patches following standard patch-clamp techniques [23]. The macro-currents were recorded in whole-cell recordings mode, in which the membrane was depolarized to various potentials for 500 ms from a holding potential of −60 mV. Mean BK current amplitude was measured at steady state, 450–490 ms after the beginning of the voltage step. The recording pipette solution routinely included in mM: 0.1 leupeptin, 12 phosphocreatine, 2 K-ATP, and 0.2 Na-GTP to prevent run-down of the Ca^2+^ current [24]. HEDTA and CaCl_2_ concentrations were adjusted to obtain the desired concentrations of free calcium, ranging from 1 to 10 mM free Ca^2+^. Free Ca^2+^ concentrations were determined by Slider’s software and confirmed with a perfectION calcium selective probe (Mettler Toledo Inc., Columbus OH). All recordings were made under symmetric K^+^ conditions where the potassium concentration was the same in the bath and recording solutions. Recording electrodes were pulled on a horizontal puller (Sutter Instruments) and fire-polished from borosilicate thin-wall capillary glass (Drummond) to a final resistance of 4–8 MΩ. Currents were recorded in voltage-clamp mode with a HEKA EPC 10 amplifier at a sampling rate of 5 kHz and 10 kHz for whole-cell and single-channel recordings respectively, and low-pass filtered at 3 and 2 kHz, respectively. Leak currents were subtracted on-line using a scaled response from a pulse without a BK current. In whole-cell mode, series resistance did not exceed 20 MΩ and was 60% compensated. Potentials and currents were digitized and stored using Patchmaster acquisition and analysis software version 2.05 (HEKA Elektronik). An agar bridge containing an Ag/AgCl pellet and 3% agar in buffer solution was used as a ground. Single-channel currents were recorded using an EPC-10 (HEKA Elektronik, Lambrecht, Germany) patch-clamp amplifier at a bandwidth of 3 kHz and were low-pass filtered at 1 kHz using an eight pole Bessel filter and sampled at 5 kHz using PatchMaster software. Single-channel conductances were obtained from I/V plots. Each patch was recorded at a given voltage from –80 to +60 for 5 s.

### Solutions

All solutions used in the patch recordings were made with doble dionized water. High-potassium pipette solution contained (in mM) 130 K_2_MeSO_4_, 2 MgCl_2_, 2 CaCl_2_, 15 HEPES. During all recording sessions, excised patches were exposed to 130 to 140 mM potassium gluconate, 0 to 4 mM HEDTA, 15 mM HEPES, 1 mM MgCl_2_, and 0.5 to 2.2 mM CaCl_2_. HEDTA and CaCl_2_ concentrations were adjusted to obtain the desired concentrations of free calcium, ranging from 1 to 10 mM free Ca^2+^. Free Ca^2+^ concentrations were determined by Slider’s software and confirmed with a perfectION calcium selective probe (Mettler Toledo Inc., Columbus OH). In these conditions, K^+^ concentrations on either side of membrane patches were the same in all experiments. Solutions were brought to pH 7.35 with Trizma base (Sigma). These conditions were optimized for seal formation and patch stability. Regular Locke’s solution contained 5 mM KCl, 145 mM NaCl, 1 mM MgCl_2_, 2.2 mM CaCl_2_, 10 mM glucose, and 10 mM HEPES, was used acclimate the dish for 20 min. prior to recording.

### Planar bilayer recording

Single channel recordings were carried out with standard planar bilayer technology. Binary lipid mixtures of DOPE with PCs (1∶1, molar ratio) or SPM (3/2, molar ratio) were initially dissolved in chloroform. The solvent was removed by evaporation with a N_2_ stream and then the dried lipid film was resuspended in decane to form a final total lipid concentration of 25 mg/ml. The bilayer was formed by painting the lipid solution across a 250 µm aperture in a Delrin bilayer chamber (model CD-P250 from Warner Instruments, Hamden CT). Bilayer capacitance was monitored by noting the current across the bilayer in response to a triangle wave (10 mV/25 ms). Membrane suspensions containing crude membrane fragments (0.2–0.5 µl) were directed to the bilayer in the *cis* chamber with a micropipette. The cytoplasmic *cis* solution contained: 300 mM KCl, 1.03 mM CaCl_2_, 1.1 mM HEDTA, 10 mM HEPES, pH 7.2. The free Ca^2+^ was measured with a Ca^2+^ electrode to be about 20 µM. Ca^2+^ standard solutions were from World Precision Instruments (Sarasota, FL). Additional drops of 0.1 M HEDTA were added to the *cis* chamber to lower free Ca^2+^ concentration so that a suitable nPo can be achieved to study the ethanol potentiation of BK channels in lipid bilayers of POPE/POPS and SPM/DOPE. The recording solutions were modified from those used in the patch experiments to obtain stable bilayer recordings and the free Ca^2+^ concentrations were chosen to get a modest channel open probability (P_o_). Alcohol was added as pure ethanol to the *cis* buffer in amounts necessary to reach the desired 50 mM concentration. Vigorous mixing of the buffer solutions was achieved by continuous stirring of both chambers with a stir bar at its full power (Sun Stir3 from Warner Instrument). The extracellular (*trans*) solution in the inner chamber contained: 150 mM KCl, 0.1 mM HEDTA, 10 mM HEPES, pH 7.2. Single channel currents were recorded with a patch-clamp amplifier (EPC-9, HEKA Elektronik, Lambrecht, Germany) [25]. The *trans* chamber was connected to ground and all voltages in the *cis* chamber were expressed relative to ground. The holding potential was usually at 20 mV unless stated otherwise. At least one minute of recording was taken as control after insertion to ensure stable channel activity before application of ethanol. Continuous recordings were taken after the application of ethanol to obtaining the time course of the ethanol response. All experiments were done at room temperature (22°C).

### Data analysis

Data for patch recordings were analyzed using Igor Pro 6 software (Wavemetrics). NPo values were calculated from all-points amplitude histograms by fitting the histogram with a sum of Gaussian functions using a Levenberg–Marquardt algorithm. Values for unitary current were obtained from the Gaussian fit of all-points amplitude histograms by measuring the distance between the modes corresponding to the closed state and the first opening level. nP_o_ as an index of steady-state channel activity was obtained from the all-points histogram and calculated from the number of channels (n) in the recording and the open channel probability (P_o_) as described elsewhere [26]. Single channel Data of bilayer recordings were analyzed with using TAC and TAC-fit programs (Bruxton Corporation). The mean open time (T_o_) was calculated from, 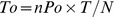 where T is the time period of the recording, and N is the number of open events. For single channel recordings, the mean open time is calculated by the sum of open-dwell time x weight of each components. The open event intervals were measured with half-amplitude threshold analysis. Data are shown as mean ± S.E.M.

## Results

### 1. Effects of β1 on steady state G-V Relationships of BK in HEK 293 cells

**Figure 2:**
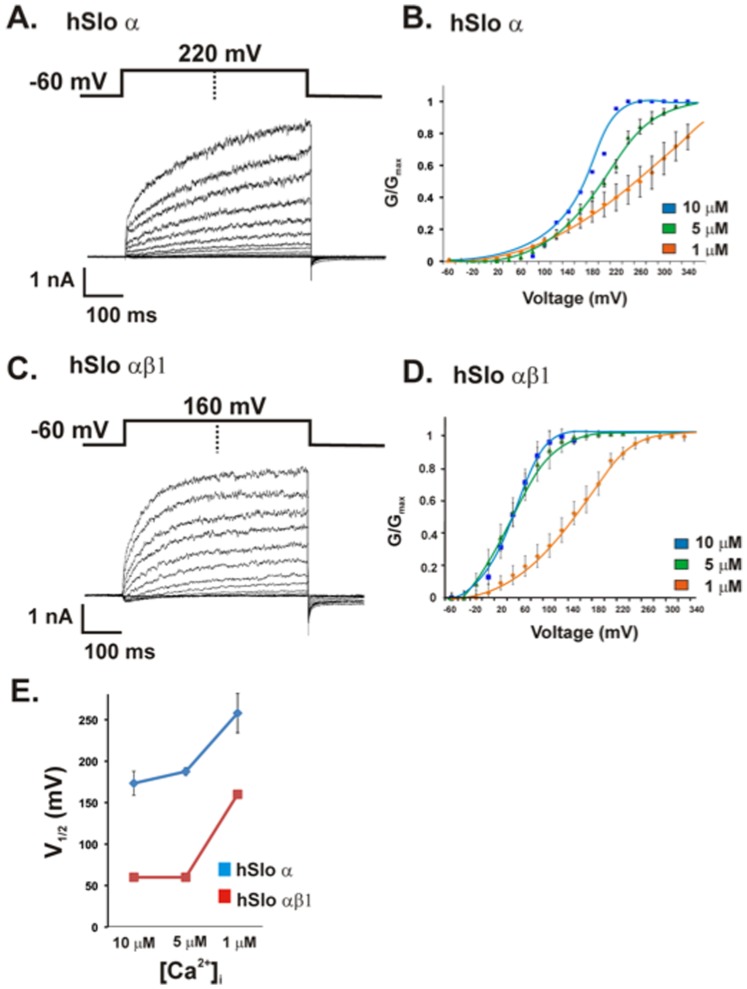
β1 creates a leftward shift in the G-V relationship of BK channels. A) The macroscopic currents recorded from HEK 293 cells stably transected with hSlo α with 5 uM Ca^2+^ inside the pipette and in the bath. The holding potential is −60 mV, traces from 0 to 220 mV with delta 20 mV. B) G-V relations determined at 1, 5, and 10 µM Ca^2+^ concentrations. C) The macroscopic currents recorded for hSlo αβ1 at 5 µM Ca^2+^. The holding potential is −60 mV, and traces from −60 to 160 mV with delta 20 mV. D) G-V relations determined at 1, 5, and 10 µM Ca^2+^ concentrations for hSlo αβ1. Each curve in B and D represents the average of between 3 and 7 individual curves. Error bars indicate SEM. E) Plots of half-maximal activation voltage (V_1/2_) vs. Ca^2+^ concentration. V1/2 were statistically different between hSlo and hSlo αβ1 for every calcium concentration tested. Error bars represent SEM, *n*  = 4–7.

We first examined the Ca^2+^ sensitivity of hSlo BK αβ1 and BK α channels stably expressed in HEK 293 cells. The macroscopic BK currents were recorded in the whole-cell patch configuration. BK currents were evoked by step depolarization at controlled intracellular Ca^2+^ of 1 µM, 5 µM, and 10 µM. Sample traces of macroscopic BK currents for hSlo α and hSlo αβ1 recorded at 5 µM free Ca^2+^ were shown in Fig. 2A and C. The co-expression of β1 subunit with hSlo α leads to the characteristic larger tail currents (Fig. 2C) compared with those of BK of hSlo α-alone (Fig. 2A). The plots of G-V constructed from current families recorded for BK hSlo α and hSlo αβ1 at different intracellular Ca^2+^ were shown in Fig. 2B and D, respectively. Increasing intracellular Ca^2+^ concentration shift the plots of G-V leftward to negative voltages for both BK of hSlo α and hSlo αβ1, however, co-expression of β1 with α subunits left-shift the G-V curves more than the BK in the absence of β1 subunits (see Fig. 2 B and D), indicating that co-expression of β1 with α subunits increases the apparent Ca^2+^ sensitivity of the channel [4], [8], [9], [12]. This is consistent with the results seen in mSlo BK channels [7]. The plot of half-maximal activation voltage (V_1/2_) vs [Ca^2+^]_i_ (Fig. 2E) showed clearly that the left-shift of activation voltage for BK in the presence of β1 subunit is Ca^2+^ concentration dependent in the range of 1–10 µM as we tested. The V_½_ (mV) for hSlo α at [Ca^2+^]_i_ of 10, 5, and 1 µM are 173.3±14.5, 187.5±4.8 and 258.0±23.7, respectively, in comparison with the V_½_ (mV), 60.0±12.8, 62±13.8, and 164±9.8, respectively for hSlo αβ1 at same [Ca^2+^]_i_. The smallest left-shift was seen at 1 µM [Ca^2+^]_i_. Similar results had been seen for mSlo BK transfected into HEK 293cells [12].

### 2. β1 increases the apparent Ca^2+^ sensitivity of BK reconstituted in negatively charged bilayers, but not in neutral lipid bilayers

To examine how the physical properties of the lipid bilayer affect β1 modulation of BK channels, we have chosen two series of lipids to make the bilayer. The molecular structures of lipids used in this study are shown in Fig. 1. The first set of lipids is composed of POPE, POPC, POPA, POPG, and POPS (left panel), which have identical acyl chains to maintain similar bilayer thickness, but have different polar headgroups, resulting in different surface charge. Among them, POPE and POPC are neutral lipids, while POPS, POPA and POPG are negatively charged. The second set of lipids is a series of phosphatidylcholines (PCs) with identical polar headgroups but with an increase of acyl chain length from C 14∶1 to 24∶1 (right panel). This will maintain a neutral surface charge but increase the thickness of lipid bilayers.

**Figure 3:**
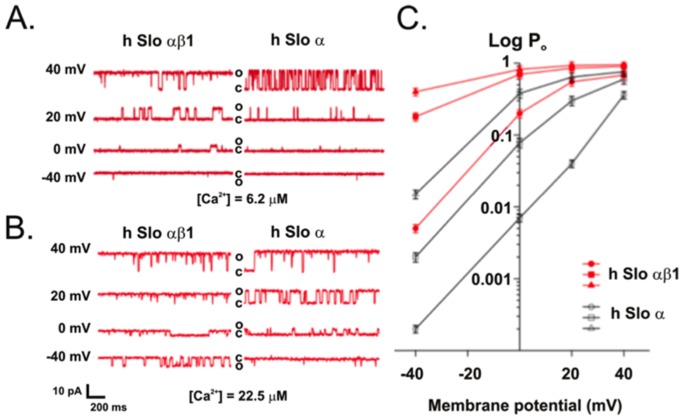
β1 increases the apparent Ca^2+^ sensitivity of the BK channel in a POPE/POPS bilayer. A, Single channel current traces of hSlo α and hSlo αβ1 recorded in low [Ca^2+^] of 6.2 µM under varied holding potentials from −40 mV to +40 mV, show an increase in channel open probability (Po) through an increase in channel open durations after co-expression of αβ1 subunit. B, Single channel currents of hSlo α and hSlo αβ1 recorded at high [Ca^2+^] of 22.5 µM under varied holding potentials from −40 mV to +40 mV, show a decrease of voltage sensitivity after co-expression of αβ1 subunit (reduced change of Po per unit change of voltage; i.e., the slope of Po-V plot). C, plots of log Po vs V of BK α (open symbols) and BK αβ1 (filled symbols) recorded at three different [Ca^2+^] concentrations: 6.2 µM (triangles), 15.6 µM (squares) and 22.5 µM (circles). Error bars represent SEM, *n* = 5–9.

The BK α and BK αβ1 channels were isolated from HEK 293 cells and then reconstituted into lipid bilayers made from lipid mixtures of POPE with either POPC or POPS, POPA, POPG in molar ratio of 3∶1. First, we reconstituted BK α and BK αβ1 into bilayer of POPE/POPS, a lipid mixture had been widely used before for studies on the function and ethanol sensitivity of BK channels [27] [17], [18], [22], [28]. Single channel activities for BK α and BK αβ1 were recorded under identical [Ca^2+^] and at varied membrane potentials. Representative current traces of single channels of BK α and BK αβ1 recorded at two different Ca^2+^ concentrations and at varied membrane voltages are shown in Figs. 3 A and 3B. At each holding potential, the presence of β1 increased the channel open probability (Po). The plot of log Po - V curves at different Ca^2+^ is shown in Fig. 3C. Clearly, the presence of β1 left shifts the log Po-V curves at any fixed [Ca^2+^] by about −40 mV (at 6.2 µM [Ca^2+^]) or more (at high [Ca^2+^]), indicating that β1 increases the apparent Ca^2+^ sensitivity of BK in negatively charged lipid bilayers, consistent with patch data obtained in HEK 293 cells. These data demonstrate that the influence of β1 on BK channel function is retained in the simplified, reconstituted lipid bilayer.

**Figure 4:**
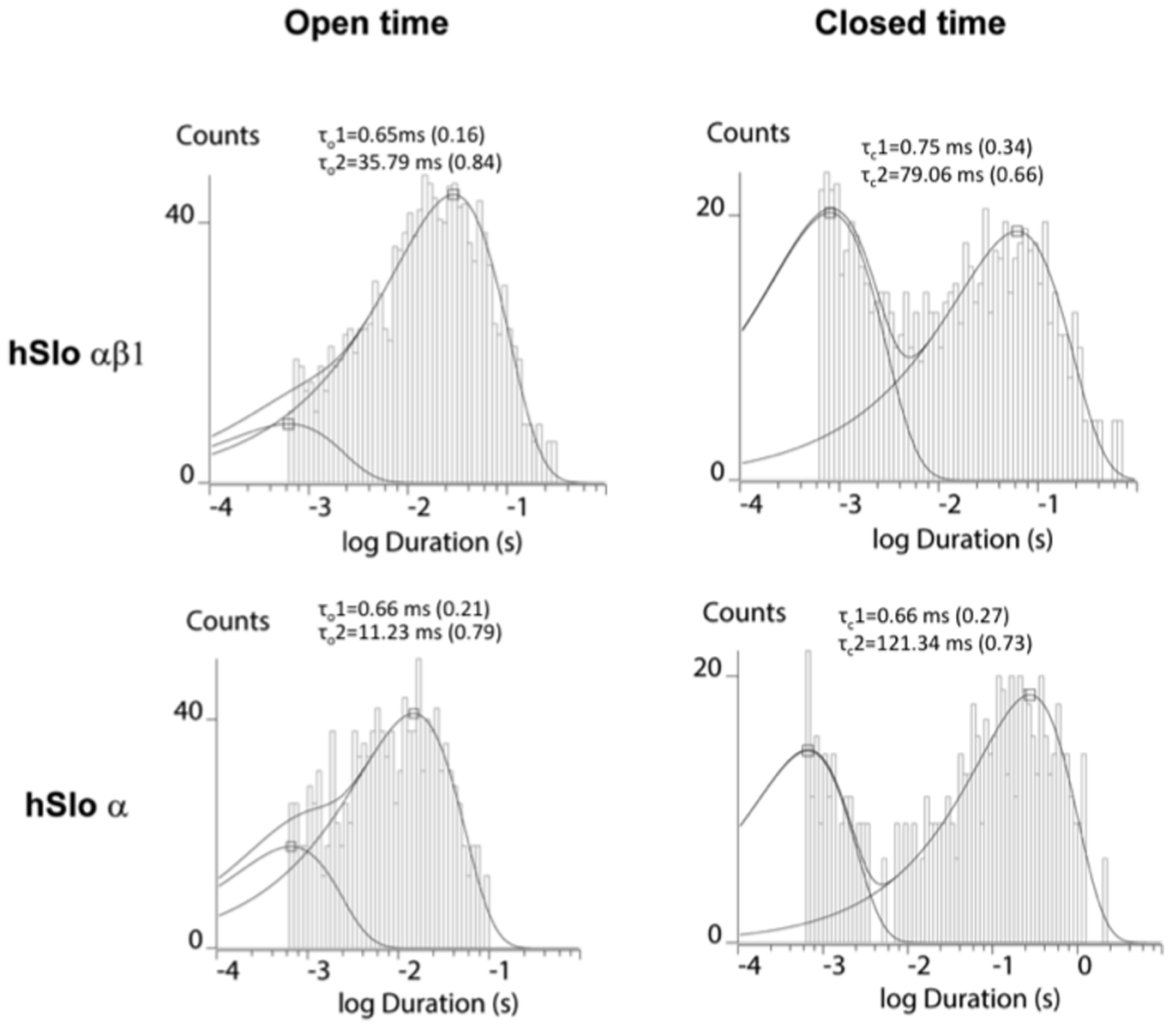
The open and closed time distribution of BK hSlo α and hSlo αβ1 recorded at free [Ca^2+^] of 6. 2 µM. Dwell-time data were plotted with a logarithmic time axis along the *abscissa* and a square-root *ordinate* exhibiting the number of events in each bin. The bin density is 20 bins per decade. A lower limit of 0.6 ms was set for the dwell time distribution of open and closed intervals, consistent with the time resolution of sampling and filtering. The time constants for each fit of the open and closed time distribution are listed in the figure, and the fractional contribution of each particular component to the composite fit is given in *parentheses*.

A close examination of the single channel gating kinetics indicates that β1 increases the channel Po mainly through an increase in open duration [3], [9]. Fig. 4 shows single channel kinetics from dwell-time histograms of BK hSlo α and hSlo αβ1 recorded under identical 6. 2 µM [Ca^2+^] in POPE/POPS bilayers. The open and closed time distributions are well fitted with two exponential functions, indicating the existence of at least two open and closed states for BK hSlo α and αβ1 in POPE/POPS bilayers. The co-expression of β1 with the α subunit did not change the number of open channel and closed states of the BK channel, rather it altered the open and closed durations and the frequency of entry into the two states. The presence of β1 increased the mean open time 2–3 fold (from 9.31±0.85 ms for hSlo α to 31.25±2.73 for hSlo αβ1, n = 3), while decreasing the mean closed time only about 50% (from 88.65±9.21 ms for hSlo α to 51.48±6.14 ms for hSlo αβ1, n = 3), suggesting that the presence of β1 stabilizes the open conformation, increasing the dwell time in the open state. Indeed, the increase of channel open duration by β1 can be readily seen in the single channel traces recorded at low 6.2 µM Ca^2+^ (Fig. 3A).

**Figure 5:**
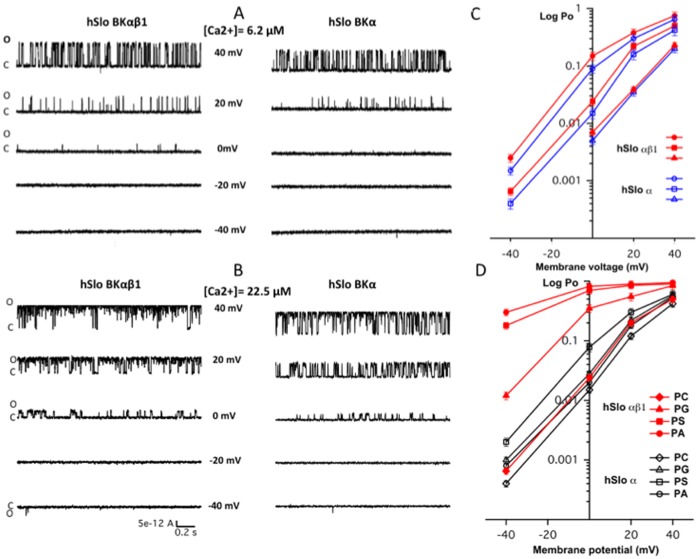
β1 has little effect on the apparent Ca^2+^ sensitivity of BK in the neutral POPE/POPC bilayer, but dramatically increases the apparent Ca^2+^ in negatively charged lipid bilayers of POPE/POPA, POPE/POPS, and POPE/POPG. A, Sample currents from a single channel of hSlo α and hSlo αβ1 recorded in POPE/POPC bilayer at low [Ca^2+^] of 6.2 µM with holding potentials from −40 mV to +40 mV. B, Sample currents of a single channel of hSlo α and hSlo αβ1 recorded in POPE/POPC bilayer at high [Ca^2+^] of 22.5 µM with varied holding potentials from −40 mV to +40 mV. C, plots of log Po vs V of BK α (open symbols) and BK αβ1 (filled symbols) recorded at three different [Ca^2+^] concentrations of 6.2 µM (triangles), 15.6 µM (squares) and 22.5 µM (circles) in POPE/POPC bilayers. These data demonstrate that β1 has little effect on the apparent Ca^2+^ sensitivity of the BK channel in POPE/POPC bilayers. D, Plots of log Po – V of BK α (open symbols) and BK αβ1 (closed symbols) in bilayers of POPE/POPC, POPE/POPG, POPE/POPS and POPE/POPA at [Ca^2+^] = 15.6 µM. Error bars represent SEM, *n* = 3–7.

We then reconstituted BK hSlo α and hSlo αβ1 into neutral lipid bilayers composed of POPE/POPC (molar ratio, 3∶1). Sample traces of a single channel of BK hSlo α or hSlo αβ1 recorded in the same free [Ca^2+^] of 6.2 or 22.5 µM at different holding potentials, are shown in Figs. 5 A and B. BK hSlo α was less active in neutral POPE/POPC bilayers than that in the negatively charged POPE/POPS bilayer, which is consistent with previously published data [19], [29], [30]. Surprisingly, the presence of the β1 subunit has little or no effect on the channel open probability in this neutral lipid bilayer. The plots of log Po – V was either not shifted, or slightly left-shifted (less than 5 mV) (Fig. 5C). Clearly, the presence of β1 did not significantly alter the apparent Ca^2+^ sensitivity in the neutral POPE/POPC bilayers, as it did in negatively charged POPE/POPS bilayers.

We next incorporated BK channels composed of hSlo α and hSlo αβ1 into POPE/POPG and POPE/POPA bilayers, both of which have a negative surface charge and examined the single channel gating. The plots of log Po-V are shown in Fig. 5D together with those of POPE/POPS and POPE/POPC at the same Ca^2+^ (15.6 µM) concentration. The data demonstrate that in three negatively charged lipid bilayers, the presence of the β1 subunit greatly increases the apparent Ca^2+^ sensitivity; the plots of Log Po – V were all left-shifted more than 20 mV, with the most voltage left-shifted in POPE/POPA bilayers, while channels in POPE/POPG bilayers displayed the least voltage left-shift (but still more than 20 mV). These data indicate that the effect of β1 subunit on the apparent Ca^2+^ sensitivity is closely related to the surface charges of adjacent lipids. In the neutral POPE/POPC bilayer, β1 has little effect on apparent Ca^2+^ sensitivity of the BK channel.

### 3. The presence of β1 reduces BK activity in thin bilayers (PC 14∶1) and thick bilayers (SPM), but has no significant effect on channel activity of BK in PC 18∶1, PC 22∶1 and PC 24∶1 bilayers

**Figure 6:**
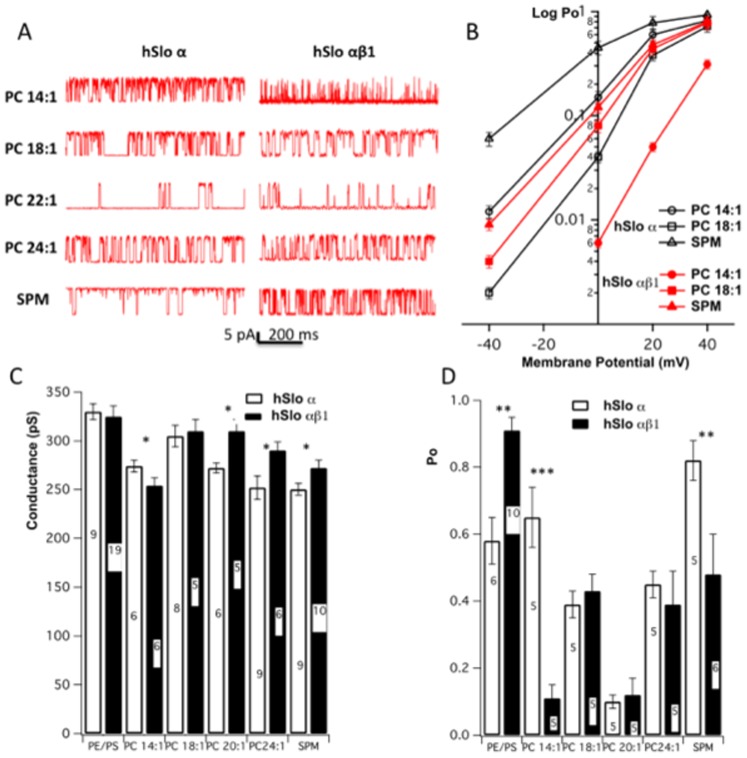
β1 dramatically affects the gating of BK in thin bilayers of DOPE/PC 14∶1 and thick bilayer of DOPE/SPM, but has little effect on gating of channel in bilayers of DOPE/PC 18∶1, DOPE/PC22∶1 and DOPE/PC24∶1. A. Sample traces of single channel currents of BK α and BK αβ1 in each bilayer. B. Plots of log Po – V for BK α (open symbols) and BK αβ1 (closed symbols) in bilayers of DOPE/PC 14∶1, DOPE/PC 18∶1 and DOPE/SPM. Error bars represent SEM, *n* = 3–5. C & D. The single channel slope conductance and open probability (Po) for BK α and BK αβ1 in bilayers of POPE/POPS, DOPE/PC14∶1, DOPE/PC18∶1, DOPE/PC 22∶1, DOPE/PC24∶1, and DOPE/SPM. The numbers of experiments are indicated in each column. All data are means ± S.E. *(*p*<0.05); ** (*p*<0.01); *** (*p*<0.001) indicate BK hSlo αβ1 different from hSlo α.

The single channel conductance and gating of hSlo α BK channels are regulated by the thickness of lipid bilayers [17], [18]. To explore the influence of bilayer thickness on β1 modification of BK gating, we reconstituted BK hSlo α and hSlo αβ1 into lipid bilayers made from lipid mixtures of equal molar DOPE with phosphatidylcholines (PCs) with acyl chain lengths of C 14∶1, C 18∶1, C 20∶1 and C 24∶1, and DOPE with SPM (which has a polar headgroup identical to PC). This series of bilayers systematically increases bilayer thickness from 55.9 to 71.1 A [18]. Sample traces of single channel current for BK composed of hSlo α and hSlo αβ1 recorded in each bilayer are shown in Fig. 6A.

The gating of BK hSlo α alone follows a V-shaped relationship, with increasing thickness of the lipid bilayer; i.e. the channel was most active in thin PC 14∶1 and thick SPM bilayers [18]. Increase in bilayer thickness leads to a decrease in channel activity from PC 14∶1 to PC 22∶1, and while the further increase in thickness results in activation of BK due to hydrophobic mismatch [18]. In the presence of the β1 subunit, however, the channel was the most inactive in thin PC 14∶1 bilayers. The single channel open probability (Po) was reduced dramatically from 0.65 for BK hSlo α-alone to 0.12 for BK hSlo αβ1. Channel openings are of the flickering type, with a very short dwell time during each open period. On the other hand, the presence of β1 has no significant effect on channel gating in PC 18∶1, PC 22∶1 as well as PC 24∶1 bilayers (see Fig. 6D). In thick SPM bilayers, where BK hSlo α-alone displayed robust gating activity, the presence of β1 subunit reduced the channel open probability significantly, although the reduction was not as dramatic as that seen in thin PC 14∶1 bilayers. The Po was reduced from an average 0.8 for BK hSlo α to about 0.5 for BK hSlo αβ1 (Fig. 6D).

Plots of log Po vs voltage for BKs composed of hSlo α or hSlo αβ1 in PC 14∶1, PC 18∶1 and SPM are shown in Fig. 6B. Clearly, the presence of β1 in these neutral lipid bilayers did not lead to a leftward shift of Po-V curves; rather, they were significantly right-shifted in PC 14∶1 and SPM bilayers, while in PC 18∶1 bilayers, only a slight leftward shift was seen, reaffirming that in neutral lipid bilayers, the presence of β1 does not increase the apparent Ca^2+^ sensitivity of the channel. Coincidentally, we also obtained the single channel unitary conductance by measuring the slope of the I-V relationship for hSlo α and hSlo αβ1 in each of the bilayers (Fig. 6C). The data show that the presence of β1 does not significantly alter the single channel conductance of the channel in POPE/POPS, PC 14∶1 and PC 18∶1 bilayers, but does increase the unitary conductance of BK in PC 22∶1, PC24∶1 and SPM bilayers.

## Discussion

Smooth muscle BK channels are more sensitive to Ca^2+^, when compared with BK channels from brain and skeletal muscle. This is due to the tissue-specific expression of β1 subunit, whose presence makes the BK channel substantially more Ca^2+^ sensitive [3], [14]. β1 itself contains no Ca^2+^ binding sites [5], thus the mechanism by which β1 increases the BK’s Ca^2+^ sensitivity has drawn a number of intensive investigations [3], [4], [5], [6], [7], [8], [9], [10], [11], [12]. The conclusions reached were not conclusive and some even contradictory. For example, early studies [4], [9] showed that β1 facilitates gating of BK by acting through Ca^2+^ [4], with the Slo1 tail domains, but not the Ca^2+^ bowl, being required. This suggested that the action of β1 subunit is not directly on the Ca^2+^ binding sites, but rather is on the allosteric machinery coupling the Ca^2+^ sites to the gate [9]. A later study [5], however, showed that the presence of β1 indeed affects Ca^2+^ binding at the Ca^2+^ bowl. By using chimeras between β1 and β2, Orio and Latorre et al [15] found that the intracellular domains of β subunit play a crucial role in mediating BK gating, while a more recent study found that an extracellular domain of β1 is required for modulating the BK voltage sensor and gating [31]. These results suggest that the interactions of β1 with α are complex, and may involve many critical domains within α and β1 subunits. Further, the effect of β1 on BK gating may not be entirely from subunit-subunit interaction. The presence of β1 can also affect the lipid-protein interaction between the pore-forming α subunit and membrane lipids.

The data we present in this study show that the increase of Ca^2+^ sensitivity of the BK channel by β1 is modulated by membrane lipid. The leftward shift of the (G/Gmax) – voltage plots, which is associated with the increase in Ca^2+^ sensitivity observed for BK hSlo αβ1 in HEK 293 cells (Fig. 2D) is retained only when the BK channels were reconstituted into lipid bilayers containing negatively charged lipids, but not in the neutral lipid bilayers, suggesting that the negatively charged lipids are involved in β1 modification of BK gating. Moreover, when hSlo αβ1 channels are placed into in a series of neutral lipid bilayers that systematically increase bilayer thickness [18], our data show that the presence of β1 has little influence on the gating of BK in bilayers of DOPE/PC 18∶1, DOPE/PC 22∶1 and DOPE/PC 24∶1, where the BK channels are likely matched well with the hydrophobic thickness of these lipid bilayers. The presence of β1, however, dramatically reduces channel activities both in the thin bilayer of DOPE/PC 14∶1 and the thick bilayer of DOPE/SPM, both bilayers being mismatched with the BK channel [18]. β1 is located between the channel core of α-subunit and adjacent membrane lipids. Thus, the presence of the β1 would inevitably affect the interaction of lipids with core BK protein by altering the lateral stress and/or hydrophobic mismatch elicited from lipid bilayer. The strong reduction in channel activity in PC 14∶1 bilayers is less likely due to the change in lateral stress caused by β1, but rather, we suggest, in thin bilayer of PC 14∶1, the extracellular loop of β1 extrudes more out of the membrane surface, and can reach the pore of the channel [31], thus effectively block the channel and impair the gating. In the thick bilayer of DOPE/SPM, the gating of BK is also reduced by β1. This likely reflects that the presence of β1 reduces the force of hydrophobic mismatch between lipids and α subunit in this thick bilayer, thus rendering the gating of the channel.

The observation that the β1 effect on BK gating is observed only when the BK channel is placed into lipid bilayers containing negatively charged lipids is intriguing. It has long been noted that BK channels (α-alone) are more active and have a larger unitary conductance when they are reconstituted in negatively charged lipid bilayers compared with neutral lipid bilayers [29]. The V_1/2_ was about 30 to 40 mV left-shifted when BK channels from rat were reconstituted into a PS bilayer (negatively charged) compared with those incorporated into a PE bilayer (neutral) [29]. Consistent with this study [29], our data show that both hSlo α and hSlo αβ1 were more active in lipid bilayers containing negatively charged lipids (POPS, POPG, and POPA) than in the neutral lipid bilayer of POPE/POPC. The plots of log Po – V (see Fig. 6D) were left-shifted for both hSlo α and hSlo αβ1 in negatively charged bilayers compared with this relationship in the neutral POPE/POPC bilayer. A close examination of plots of log Po – V (Fig. 6D), however, revealed a remarkable difference between hSlo α and hSlo αβ1. For hSlo α, the leftward shiftwas generally small, in the range of 5–15 mV, while for hSlo αβ1, the leftward shift is much bigger, in the range of 25–40 mV. Thus, the presence of β1 augments the effect of negatively charged lipids on BK gating.

The activation of BK α by negatively charged lipids was initially attributed to surface charge from the negatively charged lipids, which generate an accumulation of cations (such as K+ and Ca^2+^) and depletion of anions in the aqueous phase adjacent to the membrane, according to the well-known double-layer phenomenon in lipid bilayers [29]. However, in light of new experimental data, this conclusion has been revised and two alternative mechanisms have been proposed: 1) a specific lipid-protein interaction or 2) a more general effect of membrane curvature stress [32]. Our data seem to favor the specific lipid-protein interaction interpretation. POPC is a cylindrically shaped lipid, and POPA is a cone-shaped lipid, while POPS, and POPG is somewhere in between POPC and POPA [30], [32], in terms of molecular shape. Substitution of POPC with POPS and POPA promotes negatively (concave) monolayer curvature. If the effect of negatively charged lipids on BK gating is generated through a general effect of membrane curvature stress [32], we would expect that replacement of POPC with POPS and POPA would follow the same trend on channel gating for hSlo α and αβ1. In another words, if substitution of POPC with POPS leads to increased channel activity, then substitution of POPC with POPA will generate even more activation, for both hSlo α and αβ1. However, our data show otherwise, as we can see in Fig. 6D, hSlo α channels were less active in POPE/POPA bilayers compared with that in POPE/POPS bilayers. Thus, the activation of BK by the negatively charged lipids is less likely due to a general effect of membrane curvature stress.

Lipid involvement in K+ channel function *via* a specific lipid-protein interaction has gained more support from recent crystal structure studies of voltage-gated potassium channels [33], [34], [35]. Four phospholipid molecules (one per protein monomer), identified as PG, have been identified within the crystal structure of the homotetrameric KcsA channel [33]. The negatively charged lipid head group of PG is thought to form close range electrostatic interactions with Arg64 and Arg89 residues of KcsA located near the outer leaflet of the bilayer. The inward rectifier K+ channel (Kir) is part of a family of K+ channels that are regulated by phosphatidylinositol 4, 5-bisphosphate (PIP2) and other anion lipids. The crystal structure of a Kir2.2 channel in complex with a short-chain derivative of PIP2 [34] revealed that PIP2 binds at an interface between the transmembrane domain (TMD) and the cytoplasmic domain (CTD). More importantly, the PIP2-binding site consists of a general conserved non-specific phospholipid-binding region in the TMD and a specific phosphatidylinositol-binding region in the CTD. Therefore, negatively charged lipids can have specific interactions with protein domains from both extracellular and intracellular sides of the membrane.

In summary, our data demonstrate that the effect of β1 on the gating of the BK channel is modulated by membrane lipid. The increase of Ca^2+^ sensitivity of the BK channel by β1 involves specific lipid-protein interaction between negatively charged lipid and pore-forming α subunit. The presence of β1 augments the effect of negatively charged lipid on the gating of the BK channel. In neutral bilayers, β1 does not increase channel activity, but reduces the channel activities in the thin and thick bilayers. Thus β1 fine-tunes the channel gating not just through direct subunit-subunit interaction but also through lipid-protein interactions.
